# Improve individual treatment by comparing treatment benefits: cancer artificial intelligence survival analysis system for cervical carcinoma

**DOI:** 10.1186/s12967-022-03491-8

**Published:** 2022-06-28

**Authors:** Jieyi Liang, Tingshan He, Hong Li, Xueqing Guo, Zhiqiao Zhang

**Affiliations:** 1 Department of Gynaecology, Shunde Hospital, Southern Medical University, Shunde, 528303 Guangdong China; 2 Department of Infectious Diseases, Shunde Hospital, Southern Medical University, Shunde, 528303 Guangdong China

**Keywords:** Cervical carcinoma, Artificial intelligence, Prognostic model, Cancer specific survival

## Abstract

**Purpose:**

The current study aimed to construct a novel cancer artificial intelligence survival analysis system for predicting the individual mortality risk curves for cervical carcinoma patients receiving different treatments.

**Methods:**

Study dataset (n = 14,946) was downloaded from Surveillance Epidemiology and End Results database. Accelerated failure time algorithm, multi-task logistic regression algorithm, and Cox proportional hazard regression algorithm were used to develop prognostic models for cancer specific survival of cervical carcinoma patients.

**Results:**

Multivariate Cox regression identified stage, PM, chemotherapy, Age, PT, and radiation_surgery as independent influence factors for cervical carcinoma patients. The concordance indexes of Cox model were 0.860, 0.849, and 0.848 for 12-month, 36-month, and 60-month in model dataset, whereas it were 0.881, 0.845, and 0.841 in validation dataset. The concordance indexes of accelerated failure time model were 0.861, 0.852, and 0.851 for 12-month, 36-month, and 60-month in model dataset, whereas it were 0.882, 0.847, and 0.846 in validation dataset. The concordance indexes of multi-task logistic regression model were 0.860, 0.863, and 0.861 for 12-month, 36-month, and 60-month in model dataset, whereas it were 0.880, 0.860, and 0.861 in validation dataset. Brier score indicated that these three prognostic models have good diagnostic accuracy for cervical carcinoma patients. The current research lacked independent external validation study.

**Conclusion:**

The current study developed a novel cancer artificial intelligence survival analysis system to provide individual mortality risk predictive curves for cervical carcinoma patients based on three different artificial intelligence algorithms. Cancer artificial intelligence survival analysis system could provide mortality percentage at specific time points and explore the actual treatment benefits under different treatments in four stages, which could help patient determine the best individualized treatment. Cancer artificial intelligence survival analysis system was available at: https://zhangzhiqiao15.shinyapps.io/Tumor_Artificial_Intelligence_Survival_Analysis_System/.

**Supplementary Information:**

The online version contains supplementary material available at 10.1186/s12967-022-03491-8.

## Introduction

Cervical carcinoma (CC) was one of the most common malignant tumors in women, with 569,847 new cases and 311,365 deaths in 2018 [1]. Pathological stage was proved to be one of the most important risk factors for CC patients. It was reported that 5-year disease specific survival rates were 80% in stage I, 56% in stage II, 36% in stage III, and < 1% in stage IV [2]. Another retrospectively cohort study reported that 5-year survival rates were 95% for Stage I, 73% for Stage II, 68% for Stage III, and 19% for Stage IV [3]. The 5-year survival rates of patients receiving surgery and radiotherapy were 78.3% and 49.1% in 179 elderly cervical carcinoma patients with stage IA to stage IIB [4]. Overall, the prognosis of advanced CC patients was extremely poor with a significantly shorter life expectancy. Therefore, reliable prognostic models that could predict the prognosis of CC patients were of important clinical significance and application value.

Although radiotherapy and chemotherapy were the valuable treatments for CC patients, not all cervical cancer patients could benefit from radiotherapy and chemotherapy. A meta-analysis based on 2074 CC patients from 21 random trials provided convincing evidences for chemotherapy benefits: chemotherapy with cycle more than 14 days had a pooled HR of 1.25 (*P* = 0.005), whereas chemotherapy with cycle less than 14 days had a pooled HR of 0.83 (*P* = 0.046), suggesting that inappropriate chemotherapy cycle might reduce the survival rate of CC patients [5]. Meanwhile, neoadjuvant cisplatin with dose intensities more than 25 mg/m^2^ per week had a HR of 0.91 (*P* = 0.20), whereas neoadjuvant cisplatin dose intensities less than 25 mg/m^2^ per week had a HR of 1.35 (*P* = 0.002), indicating that inappropriate dose of chemotherapy might reduce the survival rate of CC patients [5]. The survival of patients receiving radiotherapy was poor than that of patients not receiving radiotherapy (HR = 1.09, *P* = 0.169) in 1864 CC patients [5], demonstrating that not all patients could benefit from radiotherapy. For neuroendocrine cervical carcinoma patients without lymph node metastasis, the survival of patients undergo radiotherapy was significantly poor than that of patients not undergo radiotherapy (HR = 3.36, *P* < 0.05) [6]. For stage I-IIA neuroendocrine cervical carcinoma patients with tumor size more than 4 cm, the median survival time (61 months) of patients undergo neo-adjuvant chemotherapy was shorter than that (63 months) of patients not undergo neo-adjuvant chemotherapy (*P* = 0.785) [6]. These previous studies demonstrated that not all CC patients could benefit from chemotherapy and radiotherapy, especially for CC patients with stage I and stage II.

Several previous studies developed prognostic models that could predict the prognosis of CC patients [7–10]. However, these prognostic models could only provide the survival curves for a special group, but not predict the survival curves for a specific individual patient at the individual level. Individualized survival prediction was the essential foundation of precision medicine and individualized treatment. Our research team constructed several individual mortality risk predictive tools to provide the individual mortality risk predicted curves for different cancers [11–18]. Several artificial intelligence algorithms were used to develop prognostic models for predicting the individual mortality risk predictive curves for different cancers [19, 20]. Recently, a research team from Harvard Medical School developed a novel predictive tool for predicting the individual mortality risk for glioblastoma patients based on accelerated failure time (AFT) algorithm [21]. These previous studies provided valuable ideas for artificial intelligence in predicting the individual mortality risk curves for different tumors.

Therefore, the current study aimed to construct a novel cancer artificial intelligence survival analysis system for providing the individual mortality risk predicted curves for CC patients receiving different treatments.

## Materials and methods

### Study dataset

Study dataset was downloaded from Surveillance Epidemiology and End Results (SEER) database (2010–2015). All patients were diagnosed with cervical carcinoma through pathological examination. The diagnostic criteria for cervical carcinoma was in accordance with the suggestions of American Joint Committee on Cancer (AJCC 7 edition). In order to eliminate the effects of confounding factors, living patients with survival time less than 12 months were excluded from the present study. In the study of tumor prognosis, 5 years or 10 years is the most common follow-up period for tumor prognostic study. For a well-designed prognostic study with good patient compliance, the survival time of “living patients” should be infinitely close to the longest follow-up time. The living patients with a survival time shorter than 12 months in the study dataset should consider the following two different situations: the first one is that this patient died within 12 months and can’t continue to follow up. In this case, this died patient defined as a living patient in dataset will has an adverse impact on the study conclusion, so it should be excluded from the current study accordingly. The other one is that this patient is still alive, but can’t be followed up and provide subsequent survival information due to other special reasons. In this case, the survival time of this patient is obviously underestimated, and it will has a significant adverse impact on the study result. Therefore, the living patients who were followed up for less than 12 months were excluded from the current study. Meanwhile, patients who died of causes other than cancer were excluded from the current study. All patients’ privacy information and identity information were anonymized in SEER database. All patients in SEER database signed the informed consent form at the enrollment stage. For the above reasons, ethical review and informed consent were exempted by our institutional review board. There were 14,946 cervical carcinoma patients included in the final survival analysis.

### Artificial intelligence algorithms and restricted mean survival time

Cox proportional hazard regression model algorithm was performed according to the advices in original articles [22, 23]. Accelerated failure time (AFT) algorithm was performed according to the previous studies [21, 24]. Multi-task logistic regression (MTLR) algorithm was performance in line with the suggestions of the previous articles [25, 26]. The restricted mean survival time is the sum of the areas under the survival curve in a specific time period [27–31]. As a valuable prognostic index, restricted mean survival time was widely applied to different prognostic studies [27–31].

### Statistical analyses

Statistical analyses were carried out by SPSS Statistics 21.0 (SPSS Inc., USA). Artificial intelligence algorithms were performed through R software 3.6.0 and Python language 3.7.2 according to previous studies [11–18]. *P* value < 0.05 was defined as significant statistical difference.

## Results

### Study cohort

The current study finally enrolled 14,946 eligible cervical cancer patients. The enrolled patients were randomly divided into model dataset (n = 7536) and validation dataset (n = 7410). The baseline characteristics of patients in model dataset and validation dataset were shown in Table 1.

**Table 1:** Baseline characteristics of patients in model group and validation group

Variable	Model group	Validation group	Group difference	
	N = 7536	N = 7410	Test value	*P* value
Overall survival [month]	32 (17.54)	31 (17.54)	1.822	0.177
Age [year]	48 (38.59)	47 (38.58)	2.791	0.095
Death [n (%)]	1846 (24.5)	1791 (24.2)	0.198	0.656
PT 0 [n (%)]	1 (0)	6 (0.1)	1.42	0.233
PT 1 [n (%)]	4326 (58.8)	4341 (59.0)		
PT 2 [n (%)]	1782 (24.2)	1643 (22.3)		
PT 3 [n (%)]	1158 (15.7)	1171 (15.9)		
PT 4 [n (%)]	269 (3.7)	249 (3.4)		
PN 1 [n (%)]	2018 (26.8)	1913 (25.8)	1.733	0.188
PM 1 [n (%)]	875 (11.6)	836 (11.3)	0.367	0.545
Stage 1 [n (%)]	3696 (49.9)	3708 (50.0)	1.43	0.232
Stage 2 [n (%)]	1057 (14.3)	1014 (13.7)		
Stage 3 [n (%)]	1764 (23.8)	1721 (23.2)		
Stage 4 [n (%)]	1019 (13.8)	967 (13.0)		
Radiation_Surgery [n (%)]	1971 (24.8)	1872 (25.3)	0.355	0.551
Chemotherapy [n (%)]	3937 (52.2)	3826 (51.6)	0.532	0.466

Continuous variables were expressed as mean ± standard deviation or median (first quartile, third quartile) as appropriate

### Variable importance assessment

**Fig. 1:**
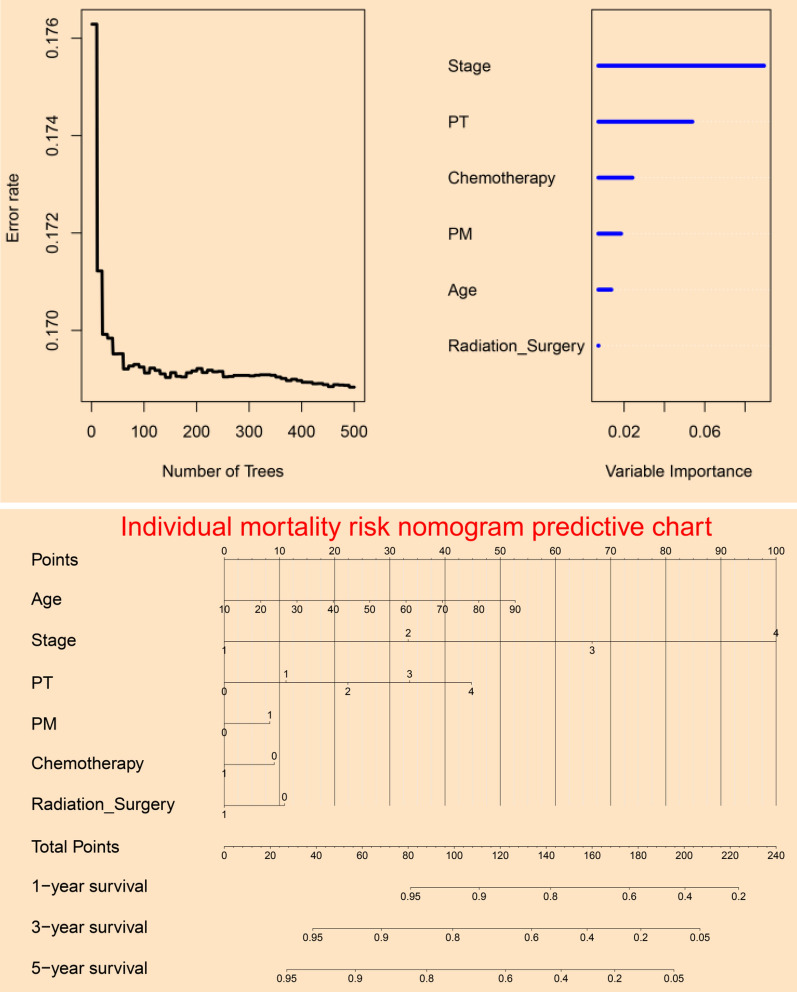
Variable selection information of predictive model: **A** error rate chart of random survival forest; **B** variable importance assessment chart of random survival forest; **C** prognostic nomogram chart generated by multivariable Cox survival regression

The current study performed random survival forest algorithm to evaluate the variable importance and explore the error rate with different number of trees. Error rate chart assessed by Out-Of-Bag method was presented in Fig. 1A. Figure 1B listed the most important variables on survival outcome from high to low: stage, PT, chemotherapy, PM, age, and radiation_surgery. Multivariable Cox regression identified stage, PM, chemotherapy, age, PT, and radiation_surgery as independent prognostic factors for cancer specific survival (CSS) of cervical carcinoma in Table 2.

**Table 2:** Model accuracy evaluation based on bootstrap resampling method

Model	Dataset	Number	C-index	C-index	C-index	Brier-score	Brier-score	Brier-score
			12-month	36-month	60-month	12-month	36-month	60-month
Cox	Dataset1	14,946	0.822	0.822	0.822	0.077	0.124	0.133
	Dataset2	14,946	0.818	0.818	0.818	0.078	0.124	0.137
	Dataset3	14,946	0.819	0.819	0.819	0.075	0.123	0.135
	Dataset4	14,946	0.819	0.819	0.819	0.076	0.125	0.138
	Dataset5	14,946	0.823	0.823	0.823	0.075	0.122	0.130
AFT	Dataset1	14,946	0.824	0.824	0.824	0.077	0.123	0.131
	Dataset2	14,946	0.819	0.819	0.819	0.078	0.124	0.136
	Dataset3	14,946	0.821	0.821	0.821	0.076	0.123	0.133
	Dataset4	14,946	0.821	0.821	0.821	0.077	0.125	0.136
	Dataset5	14,946	0.825	0.825	0.825	0.075	0.122	0.128
MTLR	Dataset1	14,946	0.827	0.830	0.830	0.077	0.133	0.131
	Dataset2	14,946	0.822	0.824	0.825	0.078	0.124	0.137
	Dataset3	14,946	0.824	0.828	0.829	0.076	0.122	0.134
	Dataset4	14,946	0.824	0.826	0.827	0.077	0.125	0.137
	Dataset5	14,946	0.828	0.830	0.830	0.075	0.121	0.130

*MTLR* multi-task logistic regression, *AFT* accelerated failure time

### Prognostic nomogram predictive chart

A prognostic nomogram mortality risk predictive chart based on Cox regression model was presented in Fig. 1C. The prognostic score could be calculated by using the following equation: prognostic score = (0.787 * Stage) + (− 0.208 * Chemotherapy) + (0.015 * Age) + (0.299 * PT) + (− 0.263 * Radiation_Surgery) + (0.245 * PM).

### Cancer artificial intelligence survival analysis system

The current study further developed a novel Cancer artificial intelligence survival Analysis system (CAISAS) for predicting the prognosis of cervical carcinoma patients. CAISAS was developed based on six previous influence factors through Cox proportional hazard regression model algorithm, accelerated failure time model (AFT) algorithm, and Multi-task logistic regression (MTLR) algorithm. CAISAS could be freely used at: https://zhangzhiqiao15.shinyapps.io/Tumor_Artificial_Intelligence_Survival_Analysis_System/.

By six major parameters and three artificial intelligence algorithms, CAISAS could provide individual mortality risk predicted curves for a special patient under different treatments.

### Performance of Cox proportional hazard regression model

**Fig. 2:**
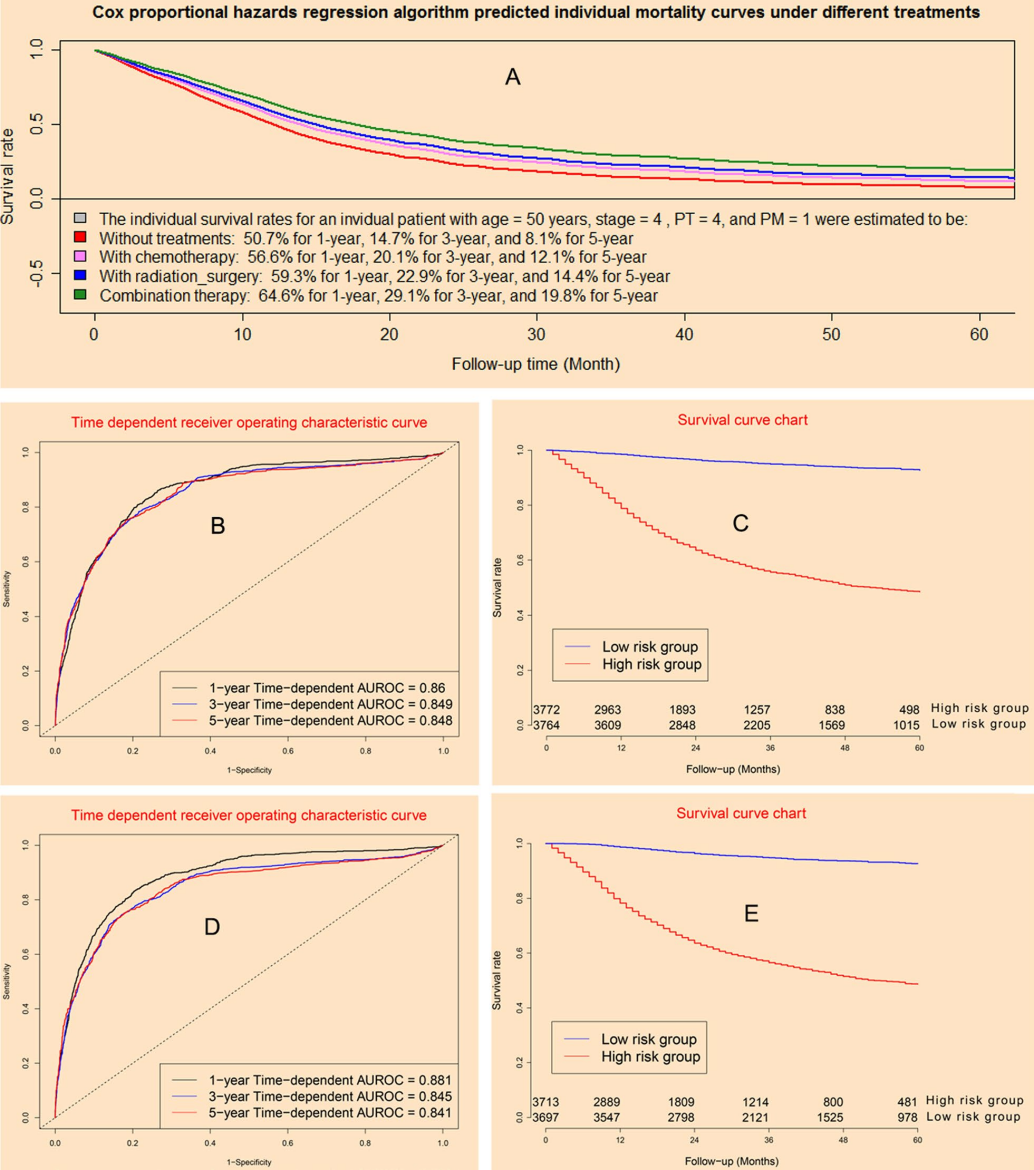
Clinical performance of Cox model: **A** predictive individual mortality risk curves under different treatments; **B** time-dependent receiver operating characteristic curves in model cohort; **C** survival curves for high risk group and low risk group in model cohort; **D** time-dependent receiver operating characteristic curves in validation cohort; **E** survival curves for high risk group and low risk group in validation cohort

Cox proportional hazard regression model could provide individual survival predicted curves for a special patient under different treatments (Fig. 2A). The concordance indexes of Cox model were 0.860, 0.849, and 0.848 for 12-month, 36-month, and 60-month in model dataset (Fig. 2B), whereas it were 0.881, 0.845, and 0.841 in validation dataset (Fig. 2D). The higher the C index, the better the diagnostic accuracy. Survival curve charts demonstrated that Cox model could discriminate high mortality risk patients from low mortality risk patients in model dataset (Fig. 2C) and validation cohort (Fig. 2E; Additional file 1).

### Performance of accelerated failure time model

**Fig. 3:**
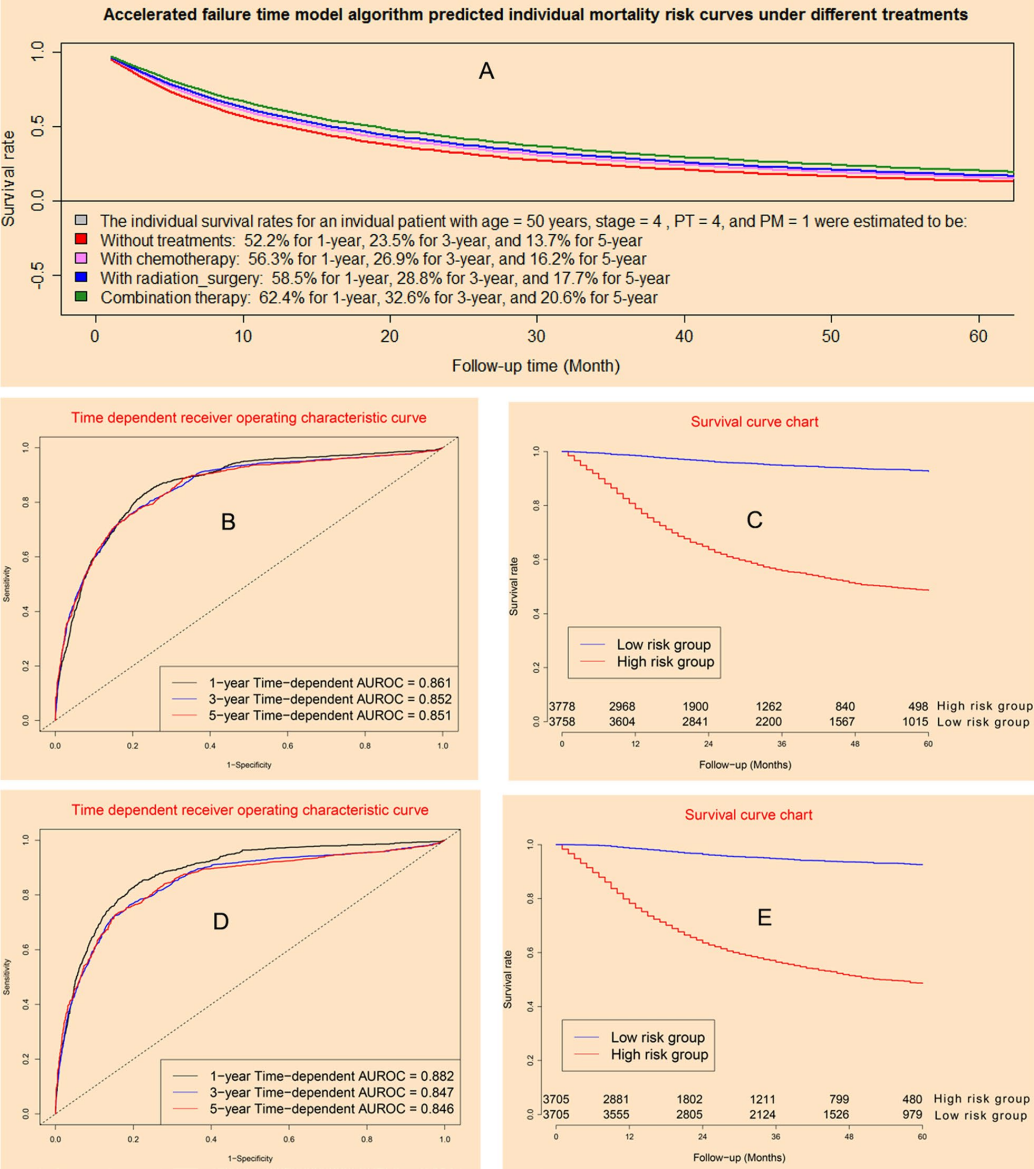
Clinical performance of accelerated failure time model: **A** predictive individual mortality risk curves under different treatments; **B** time-dependent receiver operating characteristic curves in model cohort; **C** survival curves for high risk group and low risk group in model cohort; **D** time-dependent receiver operating characteristic curves in validation cohort; **E** survival curves for high risk group and low risk group in validation cohort

Accelerated failure time model could provide individual survival predicted curves for a special patient under different treatments (Fig. 3A). The concordance indexes of AFT model were 0.861, 0.852, and 0.851 for 12-month, 36-month, and 60-month in model dataset (Fig. 3B), whereas it were 0.882, 0.847, and 0.846 in validation dataset (Fig. 3D). Survival curve charts demonstrated that AFT model could discriminate high mortality risk patients from low mortality risk patients in model dataset (Fig. 3C) and validation cohort (Fig. 3E).

### Performance of multi-task logistic regression model

**Fig. 4:**
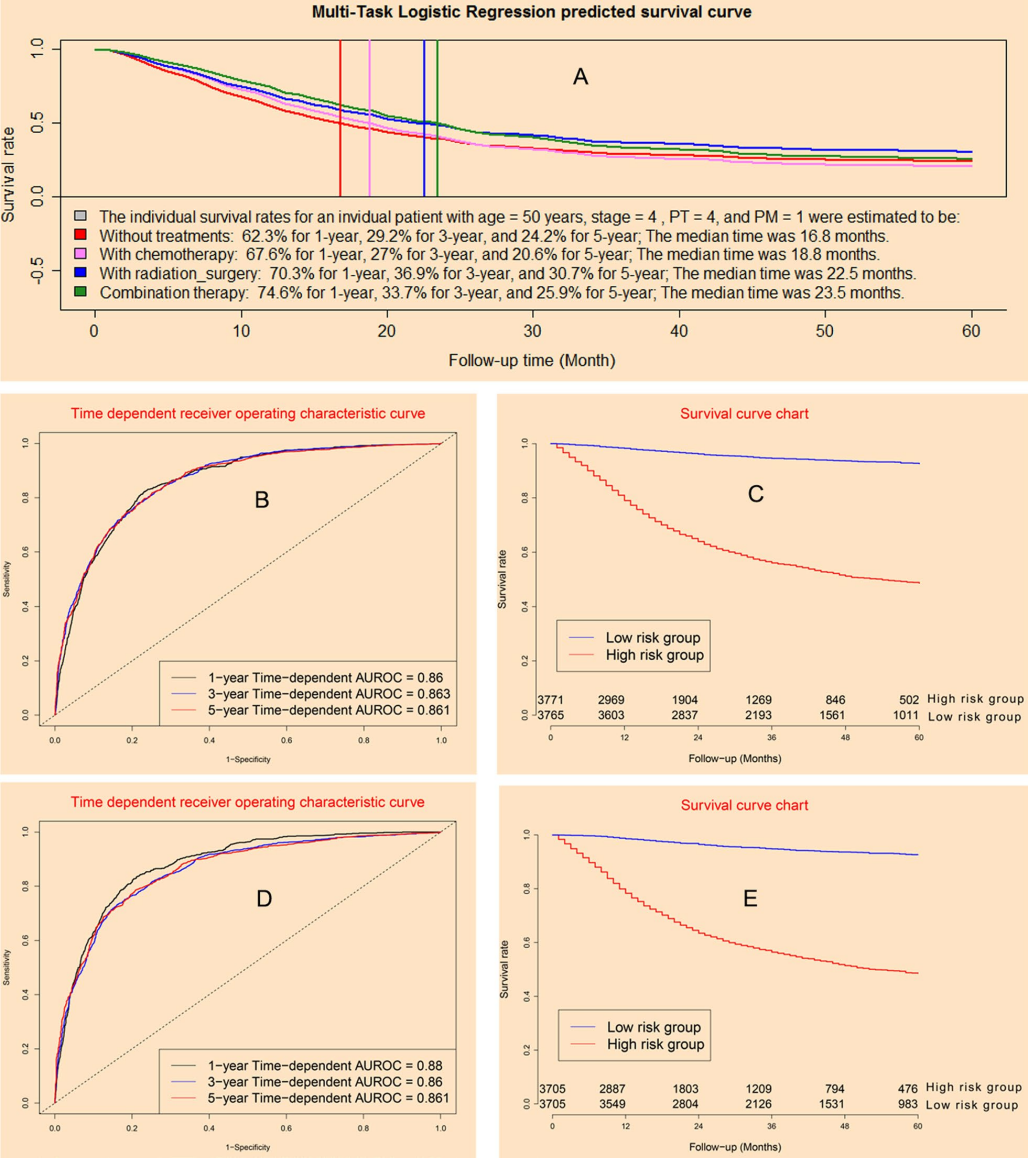
Clinical performance of multi-task logistic regression model: **A** predictive individual mortality risk curves under different treatments; **B** time-dependent receiver operating characteristic curves in model cohort; **C** survival curves for high risk group and low risk group in model cohort; **D** time-dependent receiver operating characteristic curves in validation cohort; **E** survival curves for high risk group and low risk group in validation cohort

Multi-task logistic regression model could provide individual survival predicted curves for a special patient under different treatments (Fig. 4A). The concordance indexes of MTLR model were 0.860, 0.863, and 0.861 for 12-month, 36-month, and 60-month in model dataset (Fig. 4B), whereas it were 0.880, 0.860, and 0.861 in validation dataset (Fig. 4D). Survival curve charts demonstrated that MTLR model could discriminate high mortality risk patients from low mortality risk patients in model dataset (Fig. 4C) and validation cohort (Fig. 4E).

### Brier score assessment

The lower the Brier score, the more consistent the predicted results with the actual results. Brier scores of Cox model were 0.126, 0.127, and 0.137 in model dataset, whereas it were 0.116, 0.125, and 0.134 in validation dataset for 12-month, 36-month, and 60-month, respectively. Brier scores of AFT model were 0.133, 0.128, and 0.136 in model dataset, whereas it were 0.124, 0.126, and 0.133 in validation dataset for 12-month, 36-month, and 60-month, respectively. Brier scores of MTLR model were 0.124, 0.126, and 0.137 in model dataset, whereas it were 0.116, 0.124, and 0.133 in validation dataset for 12-month, 36-month, and 60-month, respectively.

### Internal validation by bootstrap resampling method

Limited by the special requirements for chemotherapy information and radiotherapy information, the current study failed to obtain effective external validation datasets from public databases other than SEER database. Therefore, according to the recommendations of transparent reporting of a multivariable prediction model for individual prognosis or diagnosis (TRIPOD) [32], we used the self-help guide resampling method to build different internal validation datasets for evaluating the accuracy of three prognostic models. We re-sampled 14,946 patients from the original 14,946 patients in the way of put back re-sampling to build 5 internal validation datasets. Then we used these 5 internal validation datasets to evaluate the accuracy of three predictive models (Table 2). The evaluation results showed that the C-indexes of MTLR model were the best, and its highest C-indexes of 12-month, 36-month, and 60-month were 0.828, 0.830, and 0.830 respectively, suggesting that MTLR model has the best diagnostic efficiency in three prognostic models. At the same time, Brier scores of MTLR model of 12-month, 36-month, and 60-month were 0.075, 0.121, and 0.130 respectively, showing good consistency between the actual mortality and predicted mortality predicted by MTLR model.

### Survival prediction at specific time points

**Fig. 5:**
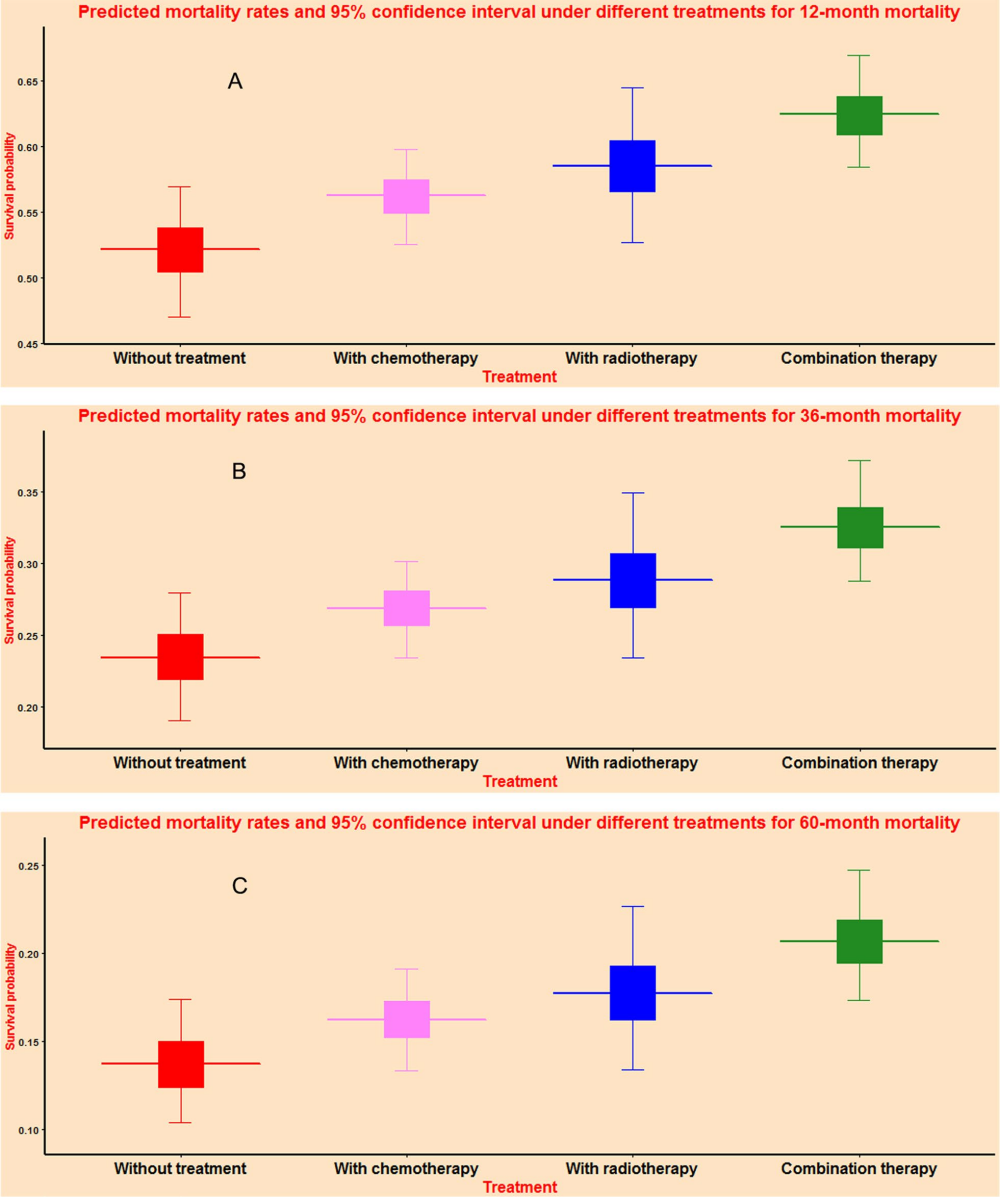
Mortality rates and 95% confidence interval by accelerated failure time algorithm for 12-month (**A**), 36-month (**B**), and 60-month (**C**)

As shown in Fig. 5, AFT algorithm provided predicted mortality percentage and 95% confidence interval at specific time points. This predictive function could provide individual mortality predicted percentage and 95% confidence interval for patients receiving different treatments at 12-month (Fig. 5A), 36-month (Fig. 5B), and 60-month (Fig. 5C). Through comparison of treatment benefits at different time points, this predictive function could provide valuable prognostic information for personalized treatment decision.

### Treatment benefits in different stages

**Fig. 6:**
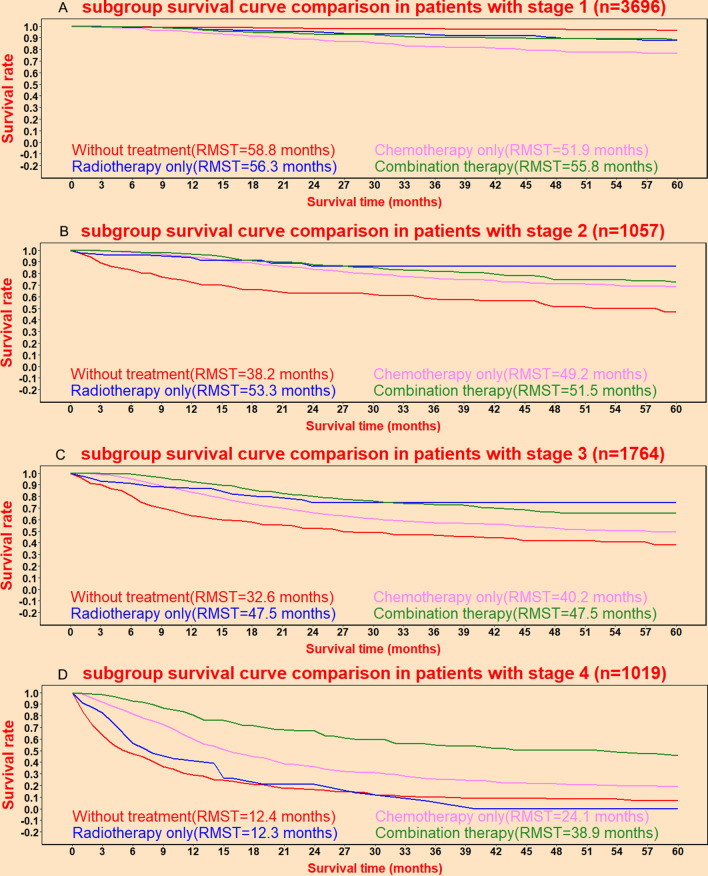
Predictive individual mortality risk curves for patients under different treatments with stage I (**A**), stage II (**B**), stage III (**C**), and stage IV (**D**)

To explore the treatment benefits in different stages, CAISAS provided predictive function in providing individual mortality risk predicted curves under different treatments in different stages. Treatment benefits under different treatments were presented in Fig. 6A for stage I, Fig. 6B for stage II, Fig. 6C for stage III, and Fig. 6D for stage IV. Figure 6B, Fig. 6C, and Fig. 6D demonstrated that radiation/surgery and chemotherapy could improve the cancer specific survival in stage II, stage III, and stage IV, whereas Fig. 6A suggested that radiation/surgery and chemotherapy did not improve the cancer specific survival in stage I. The restricted mean survival time could provide lateral prediction of survival time for tumor patients, so as to help patients better understand the survival benefits brought by different treatments. The current predictive system provided the restricted mean survival times for patients receiving various treatments in four tumor stages (Fig. 6).

### Treatment benefit of chemotherapy in different stages

To explore the treatment benefit of chemotherapy in different stages, CAISAS provided predictive function in providing individual mortality risk predicted curves for patient without chemotherapy and with chemotherapy in different stages. Treatment benefit of chemotherapy under different treatments were presented in Additional file 2: Fig. S1A for stage I, Additional file 2: Fig. S1B for stage II, Additional file 2: Fig. S1C for stage III, and Additional file 2: Fig. 1D for stage IV. As shown in Additional file 2: Fig. S1A, the survival of patients with chemotherapy was significantly poor than that of patients without chemotherapy in stage I (HR = 4.115, *P* < 0.001), whereas the survival of patients with chemotherapy was significantly higher than that of patients without chemotherapy in stage II, stage3, and stage IV, indicating that chemotherapy did not improve the cancer specific survival in stage I. The current predictive system provided the restricted mean survival times for patients receiving chemotherapy or not in four tumor stages (Additional file 2: Fig. S1).

### Treatment benefit of radiation/surgery in different stages

To explore the treatment benefit of radiation/surgery in different stages, CAISAS provided predictive function in providing individual mortality risk predicted curves for patient without radiation/surgery and with radiation/surgery in different stages. Treatment benefit of radiation/surgery under different treatments were presented in Additional file 3: Fig. S2A for stage I, Additional file 3: Fig. S2B for stage II, Additional file 3: Fig. S2C for stage III, and Additional file 3: Fig. S2D for stage IV. As shown in Additional file 3: Fig. S2A, the survival of patients with radiation/surgery was significantly poor than that of patients without radiation/surgery in stage I (HR = 2.077, *P* < 0.001), whereas the survival of patients with radiation/surgery was significantly higher than that of patients without radiation/surgery in stage II, stage 3, and stage IV, indicating that radiation/surgery did not improve the cancer specific survival in stage I. The current predictive system provided the restricted mean survival times for patients receiving radiation/surgery or not in four tumor stages (Additional file 3: Fig. S2).

### Subgroup analyses of prognostic factors in different stages

To explore the differences of prognostic factors in different stages, the current study performed multivariable Cox regression in different stages. In stage I, univariable Cox regression identified radiation/surgery and chemotherapy as risk factors for cervical carcinoma (*P* < 0.001). Multivariable Cox regression demonstrated that chemotherapy was an independent risk factor for cervical carcinoma in stage I subgroup (*P* < 0.001). For stage II subgroup, stage III subgroup, and stage IV subgroup, radiation/surgery and chemotherapy were proved to be independent protective factors for cervical carcinoma by univariable Cox regression and multivariable Cox regression (Table 3).

**Table 3:** Results of Cox regression analyses

Subgroup	Parameters	Univariate analysis				Multivariate analysis				
		HR	HR.95L	HR.95H	*P* value	Coef.	HR	HR.95L	HR.95H	*P* value
All patient (n = 14,946)	Age	1.033	1.031	1.035	< 0.001	0.015	1.016	1.013	1.018	< 0.001
	PT	2.671	2.588	2.756	< 0.001	0.299	1.348	1.286	1.414	< 0.001
	PN	3.826	3.584	4.084	< 0.001	0.044	1.044	0.964	1.131	0.285
	PM	7.288	6.798	7.814	< 0.001	0.245	1.278	1.155	1.414	< 0.001
	Stage	2.809	2.718	2.902	< 0.001	0.787	2.197	2.054	2.351	< 0.001
	Radiation/surgery	0.784	0.724	0.848	< 0.001	− 0.263	0.769	0.708	0.836	< 0.001
	Chemotherapy	2.985	2.772	3.215	< 0.001	− 0.208	0.812	0.746	0.885	< 0.001
Stage 1 (n = 7404)	Age	1.040	1.033	1.047	< 0.001	0.032	1.033	1.026	1.040	< 0.001
	PT	NR	NR	NR	NR	NR	NR	NR	NR	NR
	PN	NR	NR	NR	NR	NR	NR	NR	NR	NR
	PM	NR	NR	NR	NR	NR	NR	NR	NR	NR
	Radiation/surgery	2.259	1.842	2.770	< 0.001	− 0.046	0.955	0.757	1.206	0.699
	Chemotherapy	4.538	3.738	5.510	< 0.001	1.369	3.931	3.141	4.921	< 0.001
Stage 2 (n = 2071)	Age	1.018	1.011	1.024	< 0.001	0.015	1.015	1.008	1.022	< 0.001
	PT	NR	NR	NR	NR	NR	NR	NR	NR	NR
	PN	NR	NR	NR	NR	NR	NR	NR	NR	NR
	PM	NR	NR	NR	NR	NR	NR	NR	NR	NR
	Radiation/surgery	0.699	0.568	0.861	< 0.001	− 0.281	0.755	0.612	0.932	0.009
	Chemotherapy	0.574	0.465	0.707	< 0.001	− 0.494	0.610	0.494	0.753	< 0.001
Stage 3 (n = 3485)	Age	1.016	1.012	1.020	< 0.001	0.006	1.006	1.002	1.010	0.003
	PT	1.736	1.617	1.864	< 0.001	0.514	1.672	1.529	1.827	< 0.001
	PN	0.616	0.549	0.690	< 0.001	0.180	1.197	1.047	1.369	0.009
	PM	NR	NR	NR	NR	NR	NR	NR	NR	NR
	Radiation/surgery	0.495	0.438	0.560	< 0.001	− 0.315	0.730	0.639	0.834	< 0.001
	Chemotherapy	0.610	0.531	0.700	< 0.001	− 0.446	0.640	0.556	0.737	< 0.001
Stage 4 (n = 1986)	Age	1.014	1.010	1.018	< 0.001	0.005	1.005	1.001	1.009	< 0.001
	PT	1.172	1.114	1.233	< 0.001	0.234	1.264	1.189	1.343	< 0.001
	PN	1.028	0.919	1.149	0.633	0.009	1.009	0.899	1.132	0.882
	PM	1.219	1.045	1.422	0.012	0.610	1.841	1.539	2.202	< 0.001
	Radiation/surgery	0.437	0.374	0.511	< 0.001	− 0.621	0.537	0.458	0.631	< 0.001
	Chemotherapy	0.388	0.345	0.436	< 0.001	− 0.867	0.420	0.372	0.474	< 0.001

*HR* hazard ratio

## Discussion

Through three artificial intelligence algorithms, we developed a novel cancer artificial intelligence survival analysis system (CAISAS) for individual mortality risk prediction of CC patients. CAISAS could provide individual mortality risk prediction under different treatments through three artificial intelligence algorithms. CAISAS could provide predicted mortality percentage and 95% confidence interval for specific time points, which was helpful to display the actual treatment benefits under different treatments. Meanwhile, CAISAS provided comparison functions of treatment benefits in different stages, which were valuable to understand the treatment benefits under different treatments in different stages. Through simulating treatment benefits and individual mortality risk predicted curves for a special individual patient under different treatments, CC patient could choose the best individualized treatment.

Several previous prognostic models could predict the prognosis of CC patients [7–10], but failed to provide individual mortality risk prediction. CAISAS could not only provide the survival prediction for a specific group at the group level, but also provide the individual mortality risk prediction for a specific patient at the individual level. As far as we know, CAISAS was the first artificial intelligence survival predictive system that could provide individual mortality risk prediction for CC patients in the world.

Cox regression analysis demonstrated that chemotherapy and radiation/surgery did not improve the cancer specific survival in stage I. Previous studies provided evidences to support the result in the current study. The 3-year disease-specific survival for cervical cancer patients receiving radiotherapy and/or chemotherapy was 73.2%, which was significantly lower than 94.3% for patients receiving surgery and/or adjuvant treatment in cervical cancer patients after primary treatment [33]. Patients receiving radiotherapy only had a poor survival rate than patients not receiving radiotherapy (HR 1.48, *P* < 0.001) [34]. The overall survival in cervical cancer patients receiving radiotherapy was 53%, which was significantly lower than 61% for patients receiving conventional surgery in stage I cervical cancer patients [35]. A meta-analysis based on 2456 CC patients demonstrated that chemoradiation could improve the overall survival rate with an absolute benefit of 10% (from 60 to 70%) [36]. Chemotherapy might be not a protective factor for overall survival of stage I or II CC patients with a HR of 1.31(95% CI 0.46–3.73, *P* > 0.05) [37]. The overall survival of cervical cancer patients receiving radical hysterectomy was superior to that of patients receiving chemoradiotherapy for CC patients with stage IB–IIA [38]. These previous studies demonstrated that radiotherapy and chemotherapy might not be the best treatments for CC patients with stage I.

Cox proportional hazard regression model algorithm was used to construct predictive models for different tumors [22, 23]. Accelerated failure time model might be a credible alternative to Cox proportional hazard regression model [24, 39]. AFT algorithm was used for developing prognostic models for different cancers [40, 41]. Multi-task logistic regression algorithm was used to build predictive models for prognostic prediction [25, 42, 43]. It was reported that multi-task logistic regression model was superior to Cox model in survival prediction [44]. The concordance indexes and Brier scores of three prognostic models in the current study suggested that these three prognostic models have reliable diagnostic accuracy for prognostic prediction of CC patients.

### Limitations

First, the current study was not able to further explore the treatment benefits of specific radiotherapy, chemotherapy, and surgery because the SEER database did not provide the detailed radiotherapy, chemotherapy, and surgery information. Second, because the SEER database did not provide the information of the eighth AJCC tumor staging system, the pathological criteria was in accordance with the seventh AJCC tumor staging system in the current study. Third, in order to improve the clinical generality of CAISAS in different regions and hospitals with different medical levels, several valuable diagnostic biomarkers (such as CA242 and CA199) were not included in CAISAS. The addition of serum tumor biomarkers might be helpful to improve the predictive accuracy of the prognostic models. Fourth, CAISAS provided individualized mortality risk prediction based on the current research dataset of 14,946 cervical cancer patients. As far as the prognostic model is concerned, all individual predictive results are closely related to the clinical characteristics of the enrolled patients, so the predicted results have certain limitations and can’t represent an absolute survival predicted result, which is only for the reference of clinicians. Fifth, the current research lacked independent external validation study. Large sample size independent external validation study is very important for tumor long-term prognostic study.

In conclusion, the current study developed a novel cancer artificial intelligence survival analysis system to provide individual mortality risk predictive curves for cervical carcinoma patients based on three different artificial intelligence algorithms. Cancer artificial intelligence survival analysis system could provide mortality predicted percentage at specific time points and explore the actual treatment benefits under different treatments in different stages, which could help patient determine the best individualized treatment. Cancer artificial intelligence survival analysis system was available at: https://zhangzhiqiao15.shinyapps.io/Tumor_Artificial_Intelligence_Survival_Analysis_System/.

## Supplementary Information

**Additional file 1.** Baseline characteristics of the original study dataset.

**Additional file 2: Figure S1.** Benefits of chemotherapy for patients with stage I (A), stage II (B), stage III (C), and stage IV (D).

**Additional file 3: Figure S2.** Benefits of radiotherapy/surgery for patients with stage I (A), stage II (B), stage III (C), and stage IV (D).

## Data Availability

The study data is available at SEER database (https://seer.cancer.gov/).
