# Supplementary figures and images for: Improve individual treatment by comparing treatment benefits: cancer artificial intelligence survival analysis system for cervical carcinoma
Source: J Transl Med. 2022 Jun 28;20:293. doi: 10.1186/s12967-022-03491-8 (PMC9238034; doi:10.1186/s12967-022-03491-8)

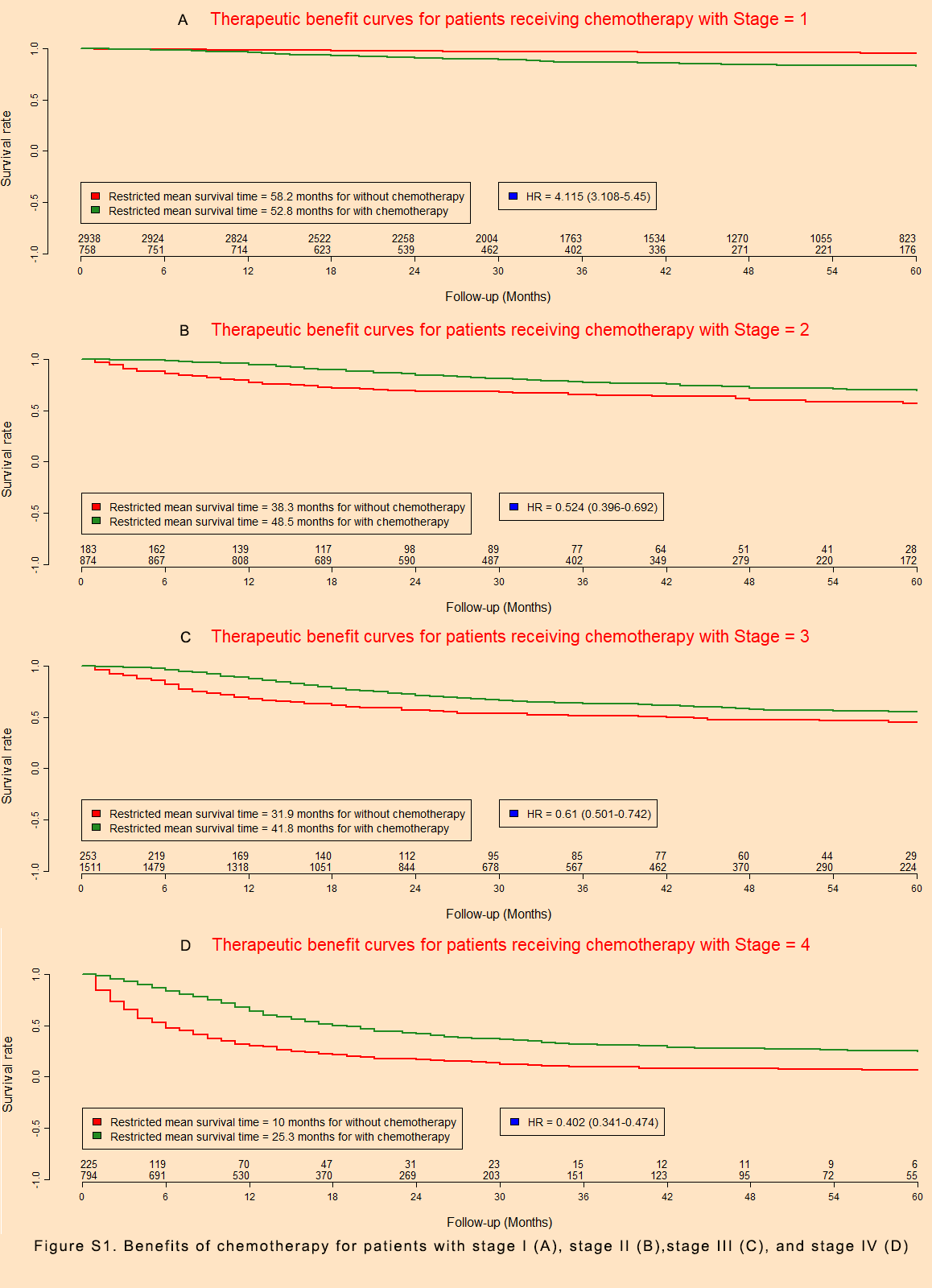

Supplement: Supplementary file 2 — Additional file 2: Figure S1. Benefits of chemotherapy for patients with stage I (A), stage II (B), stage III (C), and stage IV (D). [file 12967_2022_3491_MOESM2_ESM.tif]

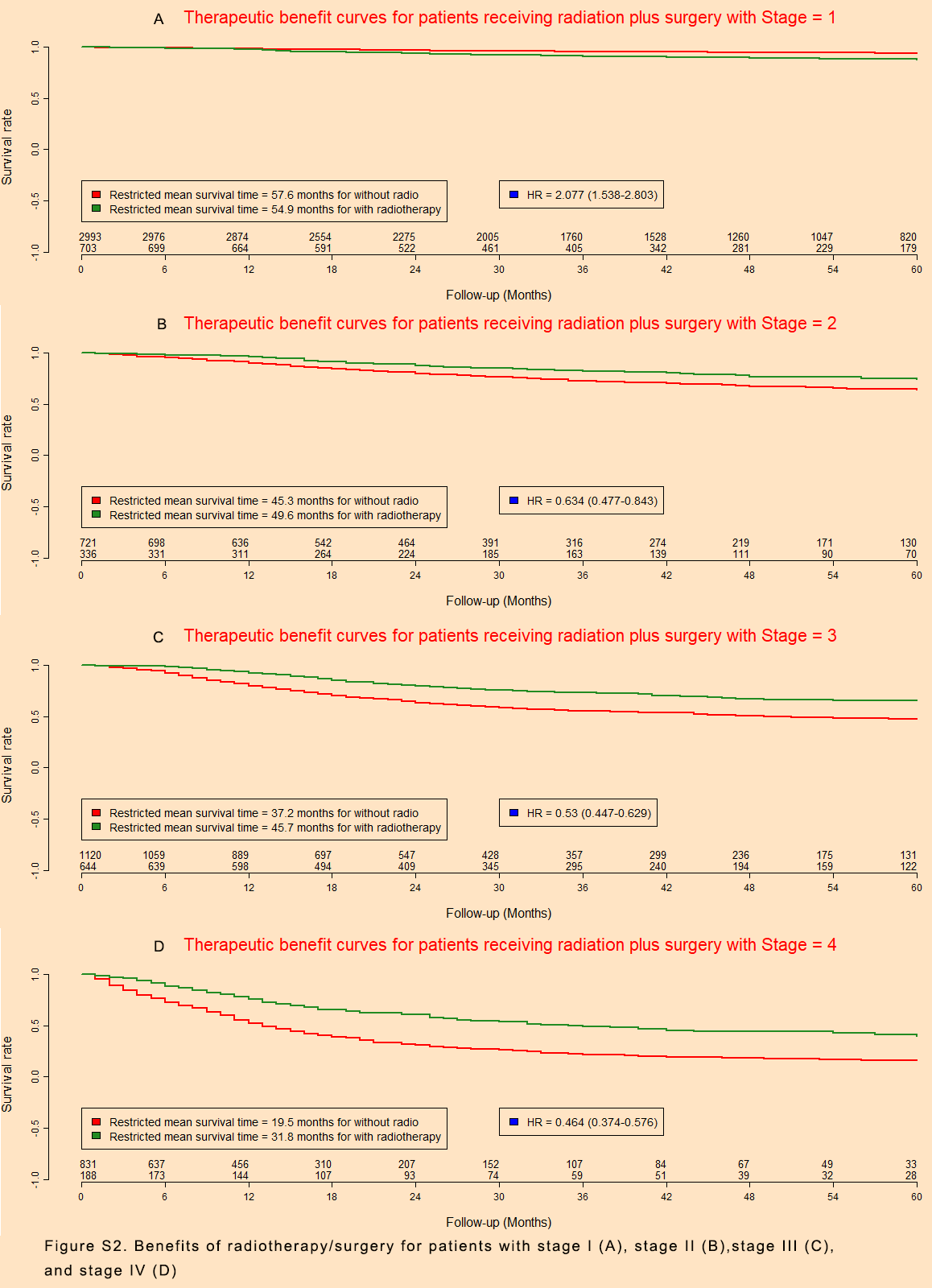

Supplement: Supplementary file 3 — Additional file 3: Figure S2. Benefits of radiotherapy/surgery for patients with stage I (A), stage II (B), stage III (C), and stage IV (D). [file 12967_2022_3491_MOESM3_ESM.tif]
